# Depression, Alcohol Abuse, and Alcoholism in One versus Two Parents and the Implications for Child Attachment and Self-Regulation in Infancy through Adolescence

**DOI:** 10.1155/2015/275649

**Published:** 2015-03-29

**Authors:** Brenda Ridgeway

**Affiliations:** City on a Hill, 529 N. New York Avenue, Liberal, KS 67901, USA

## Abstract

This study's purpose was to determine whether the influence of combined parental disorders can cause greater frequency in the occurrence of insecure child attachment and dysfunctions in self-regulation as opposed to the influence of one parent having a disorder. The research design is a quantitative meta-analysis that combined effects from 10 studies to establish differences in the frequency of occurrence for insecure child attachment and dysfunctions in self-regulation through an examination of Cohen's *d*. Global analysis of Cohen's effect (*d*) indicated that children being reared by two disordered parents had higher frequency in occurrence of insecure attachment and self-regulation dysfunction than those children reared by only one disordered parent. By addressing the issues surrounding the child population where both parents are disordered, children would have a better chance at healthy development by way of interventions that minimize the occurrence of child psychopathology and foster improvements in the social and overall human condition.

## 1. Introduction

The literature elucidated extensively how alcohol abuse and alcohol dependence create family dysfunction at every level. The implications of parental alcohol abuse and alcohol dependence for child development, relative to attachment and self-regulation, are important to child development, and just as important are the influences on child development when having a parent with a mood disorder, specifically depression. Of particular interest to this study's intent is depression and alcohol abuse and dependence. Parental alcohol abuse, alcoholism, parental depression, maternal deprivation, and parental rejection are merely a few of the devastating scenarios that a child may be raised in. Children reared in alcoholic environments strain to assemble a sense of normalcy, not really knowing how to, while fighting off daily onslaughts to their peace of mind, self-esteem, and sense of security [1]. Furthermore, an estimated 26.8 million children of alcoholics (COAs) live in the United States [2]. In fact, about one in every four children under the age of 18 is exposed to either parental alcohol abuse or dependence in their home [3]. COAs are at a much higher risk for mental health issues and long-lasting affective problems that permeate important life altering trajectories [4]. The literature revealed that depression is indicative of specific physiological mechanisms at work, or not. A study conducted by Huizink et al. (2004) [5] addressed maternal stress during the course of gestation and the possible implications to later child development. Their contention was that anomalies and overactivity of the Hypothalamic-Pituitary Adrenal (HPA) Axis in pregnant women may in fact be the pathophysiological system that precipitates psychological and behavioral pathology in the unborn children. The authors further asserted that the succession of neurochemical and hormonal responses to stress (like those noted in depression) in pregnant women negatively and perpetually transformed the central nervous system (CNS) in unborn children, therefore creating and exacerbating psychological and behavioral problems in children. Consequently, even in utero children of depressed mothers are exposed to the negative implications of the mother's depression. This further increases offspring risk for disturbances and subsequent malfunctions in the attachment relationship as well as negative implications for the self-regulation processes, respectively. For the purpose of this study attachment malfunction is defined as a* failure to function normally*. Barker [6] proposed that attachment is an emotional bond that forms between an infant and its' caregiver; the formation of the attachment bond creates the template from which other crucial tasks such as “differentiation, separation, individuation, and the internal structure of evocative object constancy” (page 1) emerge, which subsequently create our sense of self. Barker further noted that “any disturbance in the completion of those tasks can leave the child with attachment relational malfunctions or self-deficits” (page 1). The range of socioemotional and behavioral problems is broad for the children of depressed parents to include inhibitory implications on the attachment relational system Hennighausen and Lyons-Ruth [7], emotional maladjustments and disruptions in cognitive advancement [8–11]. Children reared by a depressed parent(s) are approximately at four time's greater risk to develop a mood disorder than those children of a nondepressed parent [12].

In normal circumstances, every human being is born into the world equipped with complex, yet efficient, behavioral systems ready for activation that negotiate attachment [24]. From these humble beginnings, very distinguishable and cultivated systems are evolving “that in later infancy and childhood indeed for the rest of life-mediate attachment to particular figures” (page 266). Beyond the primitive system's demands for survival, the caretaker's response to such demands has enormous impact on the child's developing mental representations of others and the mental representations of themselves [25]. Ainsworth's assessment and classification procedures used in the “strange situation” study ([26], page 932) discovered several different infant-mother attachment patterns under the umbrellas of secure and insecure attachments [27]. More recently, a fourth classification of attachment has been defined. Disorganized attachment is noted to be a mixture of anxious and avoidant and anxious and ambivalent and or resistant styles, and it is more difficult to classify than the other attachment styles [28]. These four attachment styles are in line with Bowlby's [24] premise that a child will form some type of attachment no matter what type of environment the child is subjected to relative to caregiver (usually the mother) responsiveness.

Firestone [29] concluded that disorganized attachment comes from frightening situations for a child without solutions. A disorganized attachment results from when there is no organized strategy that is workable for the child; when a caregiver's behavior is unpredictable the child has no organized strategy from which he or she is allowed to feel safe and have his or her needs met. Similarly, Waters and Valenzuela [30] discussed the hallmarks of disorganized attachment to be a result of a failure between control system components that are responsible for signal integration. Consequently, failure to pass along a signal that is either strong enough or selective enough to activate and maintain a single predominant response brings rise to disorganization in attachment. Parents that are psychologically or emotionally disturbed and/or are abusive to their child do not provide the child with a secure base support, and the unpredictability and fear experienced by the child create confusion as well as attachment instability [30].

Maternal deprivation poses very serious and profound implications for the attachment relationship between mother and child. Mullan [31] discussed this issue and noted that a child may suffer maternal deprivation even while the mother is caring for the child in terms of the mother not providing the love and emotional care necessary for healthy child development. Furthermore, Mullan also suggested that the ill effects from maternal deprivation on the attachment relationship vary and are dependent on either partial deprivation or complete deprivation. Obviously, complete deprivation would be more damaging to the child than would be partial deprivation, but partial deprivation (lack or emotional and physical availability) may create an excessive need for love, strong feelings of revenge, acute anxiety, guilt, and depression in the child. Directly related to Reactive Attachment Disorder-313.89 (RAD) is the presence of the powerful influences of child neglect and child maltreatment [32, 33]. Cicchetti [34] suggested that children who have been maltreated have been placed on a developmental trajectory that links future disruptions, failure potentials, and the mismanagement of crucial developmental transitions that may be traced back to the early attachment relationship.

Parental rejection omits most of the physical contact between parent and infant child, which is crucial for healthy secure attachment formation [35] and is the polar opposite of Bowlby's [24] notion concerning the importance of proximity between an infant and its mother during the earliest months of the infant's life. Bowlby's attachment theory provides an excellent lens through which the development of healthy personality is established, and an infant's adaptive and coping capacities play a prominent and central role in their mental health; these domains of functioning cannot be separated or understood apart from the child's attachment relationship with his or her caretaker [36]. Therefore, mental health is synonymous with early psychological antecedents that mark personality development and specificity. Bowlby [37] purported that infant proximity behavior, relative to attachment, was a modus operandi of self-regulation. Schore [38] asserted that Bowlby's attachment theory is paradoxically a self-regulation theory. The infant's request for soothing, as a proximity seeking behavior, is normally satisfied by the mothers or caretakers responsiveness, sensitivity, and attentiveness which facilitates the pattern for secure attachment so that appropriate self-regulatory processes may be developed. Secure attachment then allows the infant to sense and perceive the mother as a secure base from which exploration is safe and therefore begin to formulate autonomous self-regulation [39]. Having two parents, each with either alcohol and/or depressive disorders is the vehicle by which negative child development permeates all facets of a child's developmental life span [22]. Unfortunately, the literature was limited with respect to the vast diversity in care giving. A priori research hypotheses were as follows: (1) to explore whether there was or was not a difference in the effect for the occurrence of insecure child attachment formation when a child is reared by one parent that is either alcohol or depressive disordered versus a child that is reared by two parents each with either an alcohol disorder or a depressive disorder and (2) to explore whether there was or was not a difference in the effect for the occurrence of malfunctions in self-regulation development when a child is reared by one parent that is either alcohol or depressive disordered versus a child that is reared by two parents each with either an alcohol disorder or a depressive disorder.

## 2. Methods

The current research is a quantitative meta-analysis that explored differentiations between one versus two-parent psychopathology and their respective implications for child attachment formation and self-regulation development. Search procedures as outlined in Arthur et al. [40] were utilized to gather relevant and eligible studies. Both electronic and manual searches [40] were utilized that comprised PsycINFO, PsycARTICLES, Academic Search Premier, ProQuest, Education Research Complete, and Dissertation Abstracts databases that were utilized as search vehicles to gather published articles and unpublished research between 2002 and April 2008.

Search criteria comprised all journal articles published as a result of all possible combinations of the keywords, alcoholism, alcohol abuse, parental depression, depressive symptoms, maternal depression, child adjustment, mother-infant-child relations, internal and external behaviors, self-regulation, child emotion and affect regulation, child deviance, antisocial behavior, attachment, attachment styles, attachment security, children of alcoholics (COA), early child development, and child developmental outcomes title words. All studies that were related to parental psychopathology, child development, and child attachment and self-regulation were obtained, reviewed for relevance, and recorded for study eligibility. Each article's reference list was reviewed for additional studies and information relevant to the present research.

Additionally, selected book chapters related to the present study's interest were also reviewed for additional studies and other information. Unpublished studies were included in an effort to reduce the possibility for the “file drawer effect” ([41], page 117) and to minimize publication bias. Ultimately, this process identified a total of 125 articles that were subject area related for the current study. The resulting pool of articles was then subjected to designated inclusion criteria in order to determine the final pool of articles for inclusion in this meta-analysis. Several decisions and rules were developed and utilized in order to determine the data points that would be included in this meta-analysis. Six data points were identified and deemed appropriate for the current study and were as follows.

(1) There were no publication constraints placed on any articles researched or obtained; therefore, to be included in this meta-analytic study, articles could be either unpublished (such as an unpublished doctoral dissertation) or published. This decision is based on the notion that much more effort is needed in searching for and obtaining appropriate unpublished studies than is required for published reports' research and retrieval. One attractive component of meta-analysis is its ability to include unpublished studies; in fact, inclusion of unpublished studies was noted to reduce publication bias [41]. Furthermore, meta-analyses limited with only published studies may in fact balloon effect size estimates [42–44]; (2) at least one parent in each study had to have an existing diagnosis of alcohol abuse, alcohol dependence, or some depressive disorder (i.e., MDD, dysthymia, and depressive disorder NOS) as deemed by DSM-IV-TR or some other psychometric instrumentation for group comparisons; (3) participants in a study must have had at least one child (free from organic brain disease) between ages 2 weeks to 17 years living in their household; (4) a study had to include either an observation or psychometric measurement of child attachment security and self-regulatory processes (psychometric measure pertaining to self-regulation may include the components of self-regulation such as internalization and externalization problems and any corresponding behavioral problems, respectively, positive and negative emotionality, affect regulation and the like); (5) a study had to entail statistical data inclusive of effects or the ability for the existing data to be converted into effects, and the participant numbers for each group had to be clearly identified; (6) a study had to reveal outcome statistics (i.e., *t*, *χ* ^2^) or other salient information such as group means and standard deviations that would permit computations and conversions to the *d* statistic utilizing suitable conversion prescriptions (see [45–47]). Ten studies were deemed appropriate and thus retained for the current meta-analysis. Retention was obtained with ten (8%) of the original 125 articles. The reasons for exclusion of other articles were as follows: (a) insufficient statistical information to calculate or convert data results to a *d* statistic (31 studies or 24.4%); (b) data that were not from a primary study or were no empirical (18 studies or 14.4%); (c) use of older children (i.e., late teens, early adulthood) and adults (36 studies or 28.8%); (d) inability to locate or obtain copies of papers, articles, or studies, (seven studies or 5.6%); (e) absence of appropriate psychometric or observational measures relative to the study's variables (19 studies or 15.2%); and (f) studies that were published outside the date parameters set forth for the current study, 2002–2008, (four studies or 3.2%).

The measures from which the effects sizes were derived were categorized as child outcomes and parent outcomes. Although different authors may have used slightly different measures on the child constructs (self-regulation and attachment) and parent disorders (alcohol disorders and depressive disorders) within their respected studies, this researcher dichotomized the collective measures and categorized them into one of the variables used in the present study. For example, some authors used examples of externalizing behaviors such as anger and aggression to measure self-regulation, while other studies may have used oppositional and egotistical behaviors as the measure to the same; however, these measures fall under the construct of child self-regulation which is one of the dependent variables in the current study.

The child outcomes identified by the original authors included the following: (a) attachment style, (b) externalizing behavior problems, (d) effortful control, (e) child adjustment, (f) internalization problems, (g) cognitive and academic functioning, (h) emotional and social competence, and (i) anxiety. The parental outcomes included (a) alcohol abuse, (b) alcohol dependence, (c) major depression, (d) depressive disorder not otherwise specified, (e) dysthymia, and (f) attachment relationship to their child. The outcome data from the variables comprised in this study are discrete [48] as they show only one of two possible values, negative values, and higher degree of negative values. These values are demonstrated by way of differences in Cohen's *d* (effect size) statistic.

The reported age range of the participants was from birth to 16.10 years. Not all studies reported gender in any manner, but of those that did report gender 554 participants were females and 556 were males. A total of 2,917 participants were included in this meta-analysis; however, not all of the ten studies reported on race. Of the studies that did report on race 81.26% were Caucasian, 16.73% were African-American, 7.3% were of Other ethnic minorities, and 3% were Native American. All participants were children of parents either that had already been diagnosed with a psychopathology before the study was executed (i.e., depression or alcohol abuse/alcoholism), or that did not have a diagnosed psychopathology before the study was executed, or that had been diagnosed at the study's pretest period with a psychometric instrument (a list of imputed studies can be found in Appendix A). The standardized mean difference allowed analysis of studies that utilized various measures of attachment (secure attachment and insecure attachment and the subgroups thereof) and self-regulation (inclusive of internal and external abnormalities and malfunctions) of which findings may have been presented as standard scores, scaled scores, and T-scores but were converted so effect size estimation across studies would be possible. Cohen's *d* is favored when methodologies are not suspect in significant alterations in outcome measure variances in a nonexperimental group due to the pooled standard deviations' provision of better population estimates [43, 49]. The standardized mean difference, *d*, was the measure of effect size used in data analysis. This measure has significant utility in that it can be calculated post hoc (if necessary) from an expanded range of statistical tests (i.e., odds ratio, *t*-tests, ANOVAs, and correlation coefficients) [50]. For this study *d* was defined as

(1) $$\begin{array}{c} d=\frac{M_{1}-M_{2}}{{\mathrm{sd}}_{\text{POOLED}}}, \end{array}$$

where *M*_1_ represents the mean of the one-parent group (as shall be termed) of the *i*th study, *M*_2_ represents the mean of the two-parent comparison group of the *i*th study, and *sd*⁡_POOLED_ represents the pooled standard deviation of both groups. Rosenthal et al. [51] suggested that when the comparison is between two similar groups (i.e., two treatment groups or two groups with a similar situation) the *sd*⁡_POOLED_ from both groups is usually a more reliable estimation of the population SD and not necessarily a SD of a control group. For instance, a *d* of 0.5 indicates that the mean for one group is 0.5 SD's higher than that for the other group. This method speaks to the magnitude of the effect estimate [52]. All outcome variables (one disordered parent, two disordered parents, insecure child attachment and higher occurrence of insecure child attachment, self-regulation malformations, and higher occurrence of self-regulation malformations) were coded at pretest because pre- and posttreatment intervention comparisons were not the focus of the current meta-analysis as was the simplicity of child sample group comparisons relative to one versus two-disordered parents and child attachment and self-regulation. This can be considered a priori coding method because the coding occurred before the aggregation of effect sizes was executed [44]. As the imputed studies each yielded no less than 20 participants, Hedge's correction was not necessary.

Studies presented different data results such as means and SD, *F*-statistics, *P* values, correlation coefficients, and chi-squared values; therefore, Cohen's effect was an appropriate method for the needed conversions and standardizations for this analysis. An example of when a conversion was necessary to obtain an effect size (ES) was when one of the original study's reported data results for malfunctions in child self-regulation at child age three and child age four as *r* = 0.30 (*P* < 0.05) and *r* = 0.23 (*P* < 0.05). The formula used to obtain ES is as follows:

(2) $$\begin{array}{c} \text{ES}=\frac{N\sum x_{i}y_{i}-\sum x_{i}\sum y_{i}}{\sqrt{\left(N\sum {x_{i}}^{2}-{\left(\sum x_{i}\right)}^{2}\right)\left(N\sum {y_{i}}^{2}-{\left(\sum y_{i}\right)}^{2}\right)}}. \end{array}$$

This formula represents sample level data for the variable (self-regulation) at two different ages of the children as well as the total sample (*N*); this formula allows for conversion of both correlations (child ages) to the appropriate *d* so that collective estimation of effect sizes can be added to the aggregation across all studies. Cohen's effect size from each of the ten studies was estimated and tested for significance by using *z* test with the construction of 95% confidence interval (CI). In addition, the effect statistics for each of the 10 studies were combined and tested for global significance by using *z* test as well. The homogeneity or heterogeneity of effects from the 10 studies was tested by using the *Q*-statistic. As asserted by Lipsey and Wilson [43], the test for homogeneity is based on the *Q*-statistic and parceled out as a chi-square with *k* − 1 degrees of freedom (df), of which *k* represents the number of effect sizes [53]. Hence, the formula for *Q* is as follows:

(3) $$\begin{array}{c} Q_{\left(k-1\right)}=\frac{k\left[\mathrm{var}\left(d\right)\right]}{\mathrm{var}\left(e\right)}, \end{array}$$

where *k* represents the number of data points (*d*s, effects sizes), var(*d*) represents the variance of the sample-weighted *d*s, and var(*e*) represents the sampling error variance. The quest here was to calculate aggregate effect sizes regardless of the possible sources of heterogeneity so to bring attention to the robustness [44] of the link between insecure child attachment and malfunctions in subsequent self-regulatory processes when reared by two parents each with a psychological condition. All data were analyzed with the Statistical Analysis System, Cary, NC (SAS Program v. 9.1), utilizing interactive matrix programming (IML) procedures. Four methods of interpretation were utilized in this meta-analysis: (a) Cohen's *d*, (b) aggregation of effect sizes across studies, (c) the chi-square test, and (d) confidence intervals (CI). The global or overall effect size is a compilation of effect sizes (*d*s) from multiple studies for a particular variable being analyzed that yielded significant or insignificant effect size for all of the compiled studies. The CI explained value ranges of which a particular statistic [54] would most likely fall with a *P* value of 0.05. For this nonexperimental study, the *d* formula used was

(4) $$\begin{array}{c} d=\frac{m_{1}-m_{2}}{{\mathrm{sd}}_{\text{POOLED}}}. \end{array}$$

Any negative values of *d* indicated that the results were the opposite of what the original authors may have hypothesized in their study independently. However, despite that fact, once all 10 studies were aggregated for the current meta-analysis the effect statistics presented towards a positive direction and were statistically significant. A fixed effects model for combining effect sizes was utilized. Simply put, using a fixed effects model means that the researcher's only interest lies in the observed effect among his or her sampled studies [44]. Accordingly, Hedges and Vevea [55] contended that fixed effects meta-analyses are typically and appropriately used for making conditional inferences, which were applicable to the collection of studies used in the current meta-analysis.

## 3. Results

Because some studies addressed parental depressive disorders exclusively (one-parent psychopathology and the implications for either child attachment development or child self-regulation development while other studies addressed parental alcohol disorders exclusively and the implications to the same) they will be presented separately for better conceptualization. By presenting the results in this manner, the researcher hopes that those who read this work will remain open-minded and unbiased to this study's intent, which is to investigate and call for more research efforts towards the population of children that are being reared by two disordered parents. Although the individual percentages appeared somewhat varied, the total agreement percentage between both raters was impressive at 100% agreement. Because of the differences in sample size from each of the 10 studies comprised in the current meta-analysis, the average value of *d* was weighted to account for the differences, which allowed more weight to studies that used larger samples. By averaging these results across all 10 studies, it was possible to achieve a more reliable estimation of what is known about the current study's focus, which was insecure child attachment formation and disturbances in self-regulation development.

The studies included in this meta-analysis presented differences in data results which prompted effect size conversions and standardizations utilizing Cohen's effects. The current study utilized outcome effects for the dependent variables and did not collectively average the studies' outcome effects. Cohen's effect was an appropriate method for estimation of each study's variables and thus obtaining the appropriate effect statistics for analysis. After inspection of the *Q* statistics, it appeared that there was variability among effect sizes. As expected, the *Q* statistic for one-parent depressive disorder was significant (*Q* = 474.12, df = 4, *P* = 0.01) and with an alpha level set at 0.05 indicating that the effect sizes were heterogeneous. Similar findings were present for the rest of the variables in the sample as well (*Q* = 218.60, df = 2, and *P* = 0.01; *Q* = 7.25, df = 2, and *P* = 0.03; *Q* = 123.68, df = 3, *P* = 0.01, and *Q* = 51.14, df = 4, and *P* = 0.01, resp.). As only one study was available for one alcohol disordered parent and the implications for child attachment, *Q* statistic could not be obtained. In addition, the critical values for *χ* ^2^ with *k* − 2 (df) = 5.991, *P* = 0.05, with *k* − 3 (df) = 7.815, *P* = 0.5, and with *k* − 4 (df) = 9.488, *P* = 0.05. Interestingly, the observed *Q* statistics were notably greater than the critical values of *χ* ^2^ with a set alpha level of 0.05, and this is an indication that a relationship indeed exists between these specific parental disorders and the child constructs. The two studies included in this meta-analysis that comprised two parent psychopathology and implications to the child constructs (child attachment and child self-regulation) yielded even more impressive value differences than the former (*k* − 6 (df) = 12.592, *P* = 0.05, and *k* − 7 (df) = 14.067, *P* = 0.05). Upon inspection of the observed *χ* ^2^ (*Q* value) values, they were greater than the critical *χ* ^2^ values with alpha level set at 0.05. Normally, when indication of significant heterogeneity has been determined, the researcher may choose to examine the distribution of effect sizes for such things as outliers or moderators that may account for the variability. However, as Quintana and Minami [44] so eloquently noted that the choice to either subdivide studies into subgroups or simply omit certain studies as outliers should not be executed merely because of observed statistics (i.e., studies' effect sizes or the homogeneity statistic) or on investigating the studies' effects on the effect size distribution; in fact, there are circumstances when a significant *Q*-statistic should not prompt the researcher to stop the aggregation of effect sizes across a group of studies that are heterogeneous (Quintana and Minami). Quintana and Minami mentioned several occasions when a significant *Q*-statistic is warranted. The first would be that a significant degree of heterogeneity is present due to high statistical power that results in statistical significance where practical significance is devoid. A second occasion would be where effect sizes would differ and there may very well be no methodological or theoretical reason to justify exclusion of a study that adds to the heterogeneity. A third occasion, Shadish et al. [56] proposed that a third occasion may be that the heterogeneity is appropriate if the researcher simply wishes to execute a calculation of an aggregated effect size no matter the nature of the heterogeneity sources simply to reveal the magnitude of the relationship or the link between variables, which is the case in the present meta-analysis. Lastly, as Quintana and Minami [44] pointed out, if aggregating effect sizes is the researcher's intent then utilizing heterogeneous estimates may certainly be appropriate.

The literature was clear that parental psychopathology impacts child attachment and self-regulation [57–60]. Statistics in the current meta-analysis revealed both small and large effect sizes for child attachment and self-regulation as a result of having been reared by one parent with a psychopathology (see Table 1). The effect statistics from studies with one depressive parent are presented in Tables 13 and 14. The standardized statistics for Milgrom showed less deviation from the mean value (*M* = 14.15, *sd*⁡ = 3.90, and *N* = 162) than did Wittenborm (*M* = 107.09, *sd*⁡ = 15.45, and *N* = 75) with regard to insecure child attachment. As for the Harris study, the effect size was derived *F* statistic and no means or standard deviations were available. The CI around the mean effect size indicates a range of which the population mean is most likely to be considering the observed data. For example, for the Milgrom study, the 95% CI around the mean effect size indicates that there is a 95% probability that the population mean effect size is between the two values, in this case 0.054 to 1.00 (see Table 1).

The CI around the mean effect size speaks to the degree of precision of the estimation of that same mean effect size. Furthermore, if the CI does not include 0 then the mean effect size will certainly be statistically significant at the CIs specific range. The effects for insecure attachment from the Milgrom and Wittenborn studies were significant (*d* = 0.77, SE *d* = 0.12, 95% CI = 0.54–1.00, and *P* < 0.01; *d* = 0.38, SE *d* = 0.16, 95% CI = 0.07–0.70, and *P* < 0.02). Although the Wittenborn *d* was small, it was still significant. However, for the Harris [15] study, the effect size was not statistically significant (*d* = 0.23, SE *d* = 0.02, CI = −0.17–0.63, and *P* > 0.05) as evidenced by the 0 in the CI, which corresponds with a nonsignificant mean effect size as an independent study. The CI for a mean effect size is derived from the standard error (SE *d*) of the mean and a critical value from the *z* score distribution, for example, 2.39 for *α* = 0.05. Nonetheless, when the effect sizes for child attachment were aggregated across all three of the studies (global) the statistical significance was apparent as demonstrated in Table 2.

The effect size was moderate (*d* = 0.57) across the studies and the *P* value was less than 0.05 indicating that the effect size was significant when all the studies are combined. Although the effect size of one of the studies [15] was not significant, the overall effect size here was significant (*P* < 0.05).

**Figure 1:**
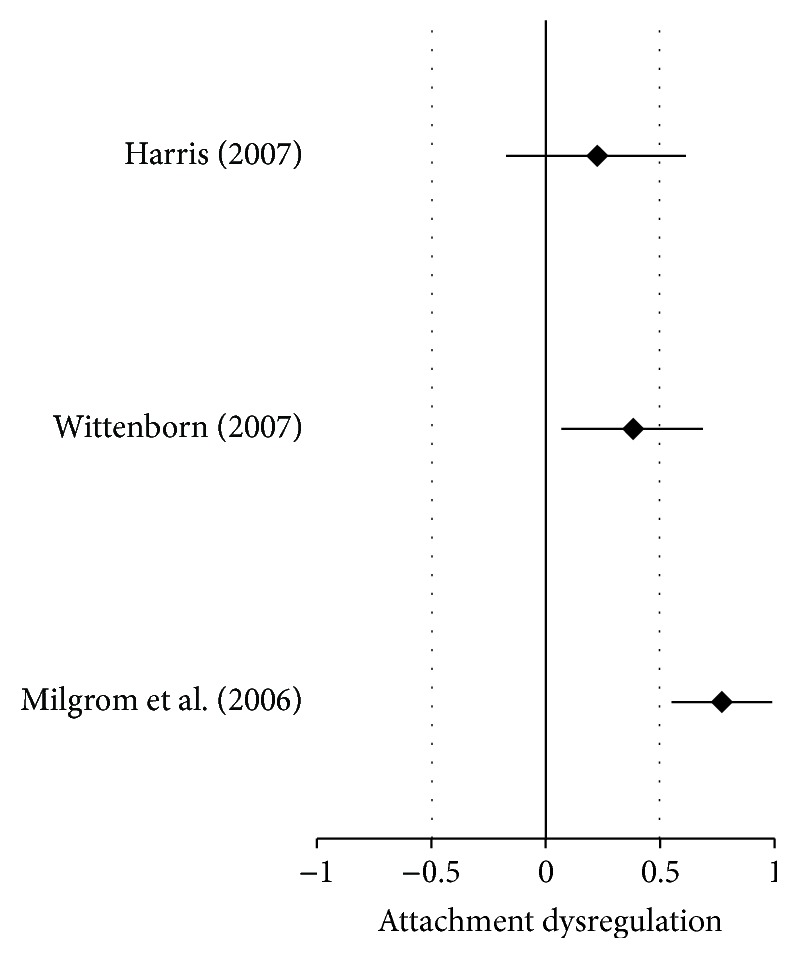
Forest plot of insecure attachment due to one depressive disordered parent.

The forest plot is one of the most attractive and convenient methods of presenting a visual aid for effect sizes from different studies. The forest plot in Figure 1 (and all forest plots included in the current study) also includes 95% CI and is an easy and straightforward way to read and interpret effect sizes from different studies. For instance, two of the studies [13, 14] presented significant effect sizes and one study [15] presented a nonsignificant effect size.

Figure 1 presents a quick visual indication of how the effects are either significant or not significant. Note how the Harris study crosses the zero line which quickly shows its nonsignificance as noted earlier. The studies that addressed malfunctions in self-regulation regarding combined disordered parents yielded larger global effect statistics than those effects for children with one disordered parent as evidenced in Tables 4 and 11. The overall global significance was also greater for malfunctions in self-regulation with combined disordered parents (ME/*g* + = 0.73, SE = 0.04, and *P* < 0.01) than the global significance for one disordered parent (ME/*g* + = 0.57, SE = 0.01, and *P* < 0.01).

**Figure 2:**
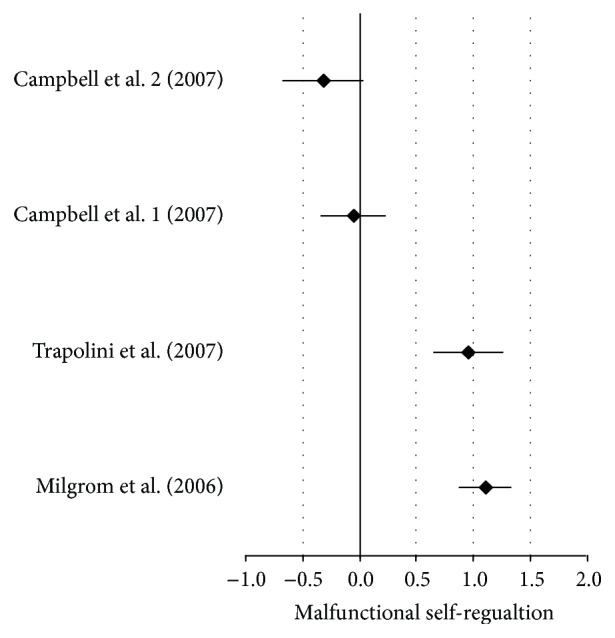
Forest plot of self-regulation malfunction from one depressive disordered parent.

Table 4 displays that the effect size was moderate (0.56) across studies. The *P* value was less than 0.05 indicating statistical significance when studies were aggregated. Although two studies presented significant effects and two studies presented nonsignificant effect statistics, once the studies were combined the effects yielded statistical significance regarding malfunctions in child self-regulation development. Visual inspection of effect statistics is displayed in Figure 2. Visually, it is easy to see the two studies that crossed the zero line that consequently rendered nonsignificant effect size statistics; however, with aggregation of the three studies' effect sizes produced a statistically significant overall effect.

Three of the included studies of this meta-analysis concerned one parent alcohol disorder and the implications to child attachment formation and child self-regulation development. Of those, one presented data on both child attachment formation and child self-regulation [18, 19] while two studies presented data only on child self-regulation malfunctions. Therefore, all three studies showed effects for self-regulation (to include El-Sheikh) and the El-Sheikh study also showed effects for insecure child attachment formation (see Tables 4 and 5). The effect size statistics from studies with one alcohol disordered parent are presented in Tables 15 and 16.

**Figure 3:**
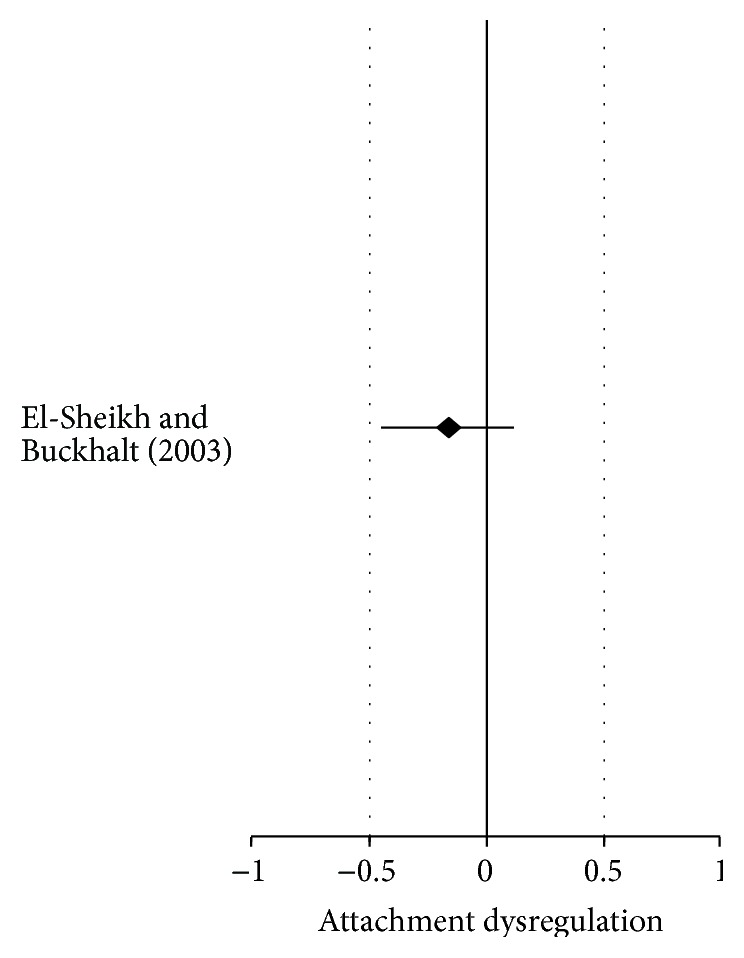
Forest plot of insecure child attachment from one study with one alcohol disordered parent.

Notice that the CI's lower and upper levels contain zero, meaning that as an independent study the effect size for insecure attachment was nonsignificant (*d* = −0.16, SE = 0.15, CI = −0.45–0.13, and *P* > 0.22). As it turned out, only one study was available regarding parental alcohol disorder and child attachment so there was nothing to compare data with and the *Q* statistic could not be obtained using only one study. However, estimates are displayed for future reference. Because only one study was available, the global test of significance was the same as for the one available study (ME/*g* + = −0.16, variance = 0.02, SE = 0.15, and *P* > 0.22) as displayed in Table 5. Consequently, visual inspection of the one study that was available shows the nonsignificance as demonstrated in Figure 3.

Table 6 shows that the Eiden et al. [20] and Kelley and Fals-Stewart [21] studies present their effects in the negative range. In addition, both of these studies' respected CIs contain zero rendering these two studies nonsignificant as independent studies. The effect size statistics from the studies with one alcohol disordered parent are located in Appendix B. The overall global test was significant despite the fact that individual studies' effects were highly variable (see Table 6), which demonstrates the power of meta-analysis, the ability to quantitatively combine different effects.

This demonstrates the utility of meta-analysis in which the whole is greater than the sum of its independent components. The effect size is a statistic that quantifies the degree to which sample data results diverge from what is expected or specified in the null hypothesis, and therefore rejects the null hypothesis of the current study [61].

**Figure 4:**
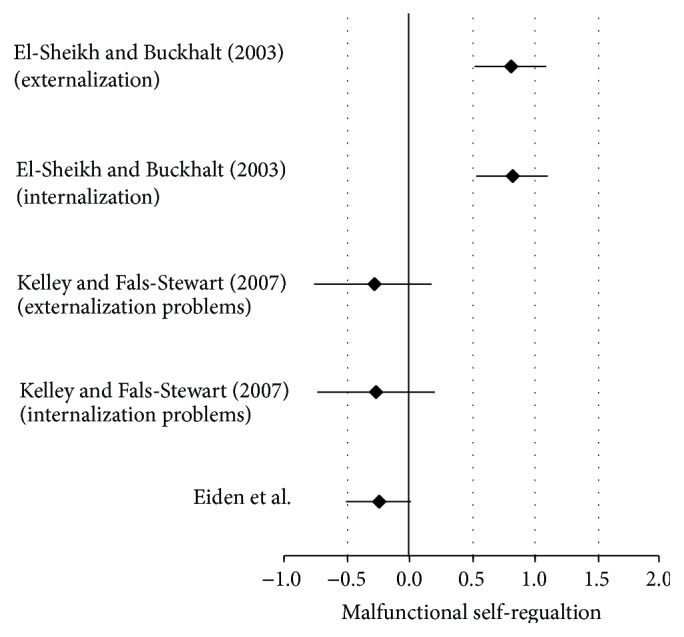
Forest plot of child self-regulation malfunctions from one alcohol disordered parent.

The one study [18, 19] shows a nonsignificant effect size for insecure attachment. However, effect statistics concerning one parent alcohol disorder and child self-regulation malfunction showed different effects outcomes even though all means were not available for conversion due to derivation of effect sizes from different statistics as indicated in Figure 4. Note the varying direction of effects, and although some effect sizes were significant some were not, and they present in the negative direction, meaning the opposite side of the zero line. The forest plot provided in Figure 4 immediately provides a meaningful visual aid to how a significant global effect was achieved once the studies were aggregated despite the studies that crossed the zero line (see Figure 4).

For instance, in the Kelley and Fals-Stewart [21] study, the authors examined preadolescent and adolescent groups (in terms of self-regulation) in the context of one alcohol disordered parent. The adolescent group yielded larger effects for malfunctions in self-regulation than the preadolescent group, even when the parent with the alcohol disorder received treatment. The author's indicated that malfunctions in adolescent self-regulation were more resistant to change than for the preadolescent group. The two studies that produced effects in the negative range were not statistically significant, as independent studies, because the CIs contained zero as shown in Table 6 (*d* = −0.24, SE −0.14, CI = −0.050–0.03, and *P* = 0.09; *d* = −0.26, SE = 0.24, CI = −0.73–0.21, and *P* = 0.22; and *d* = −0.28, SE = 0.24, CI = −0.76–0.19, and *P* = 0.20, resp.). Nonetheless, when studies were aggregated the effect was statistically significant (ME/*g* + = 0.29, var = 0.01, SE = 0.07, and *P* = 0.01). A search of the literature turned up two studies of which explored two-parent psychopathology and the implications for child self-regulation but unfortunately none on child attachment. Eiden et al. [22] and Slep and O'Leary [23] examined the implications to child self-regulation when being reared by one parent with a depressive disorder and the other parent with an alcohol disorder. All effects were statistically significant even as two of them were in the opposite direction as displayed in Table 8, while effect size statistics from the studies with combined disordered parents are shown in Tables 17 and 18.

Note that Eiden et al. [22] yielded nonsignificant effects for malfunctions in child self-regulation as indicated by the mothers report on their children's self-regulatory capabilities at child age 4 (*d* = 0.68, SE = 0.09, CI = 0.85–0.50, and *P* = 0.01, as seen in Table 8). In addition, both parent's report on their children's self-regulatory capabilities was also nonsignificant (*d* = 0.75, SE = 0.09, CI = −0.92–0.57, and *P* = 0.01) at child age 4 as opposed to the parental reports at child age 3. Accordingly, these two studies were included in the aggregation of all 10 studies used to demonstrate the projective scenario of combined parental psychopathology and their respected implications to child attachment formation and self-regulation development that follows. It is the hope of this researcher that by announcing the two combined studies first, as shown above, it will help the reader with conceptualization and limit confusion. The global effect however was statistically significant (ME/*g* + = 0.32, var = 0.00, SE = 0.03, and *P* = 0.01) as shown in Table 8.

The reliability of the mother's report and both parent's report may have in fact been influenced by their respected disorders and must be taken under consideration when interpreting these data results. Equally important is that mothers and both parents reports may have also been influenced by a laundry list of possibilities such as changes in SES over the one year span, relationship, employment, or any one or several exacerbating medical, mental, or biosocial occurrences as well.

**Figure 5:**
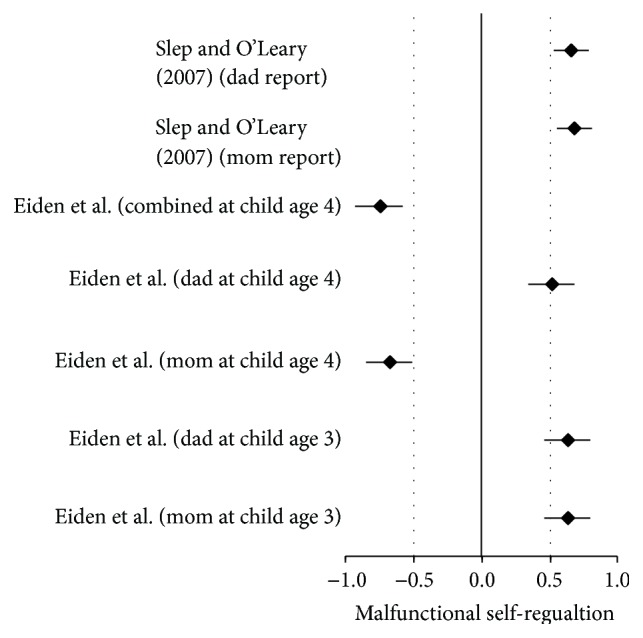
Forest plot of malfunction in child self-regulation for the two studies with combined parental psychopathology.

Overall, the effect size across the two studies was statistically significant despite that a few effects went in the opposite direction (see Figure 5) as visual inspection of forest plot demonstrates. Note how moms' report in Eiden et al. [22] crosses the zero line on the negative side at child age 4 (*d* = −0.68, SE = −0.09, CI = 0.85–0.50, and *P* = 0.01) but not the dads' report at child age 4 (*d* = 0.52, SE = 0.09, CI = 0.34–0.69, and *P* = 0.01), meaning that moms' report effect statistic is nonsignificant at child age 4 but dad's is significant. Worth noting is that the dads' report at child age 4 was significant (*d* = 0.52, SE 0.09, CI = 0.34–0.69, and *P* = 0.01), but when the parents' provided combined reports at the same child age (4), suddenly the dads' report became nonsignificant as well when it was combined with moms' report (*d* = −0.75, SE = 0.09, CI = −0.92–0.57, and *P* = 0.01). Perhaps here, moms may have had some influence on the dads report on their child's self-regulatory capabilities; however, at child age 3 it is just the opposite showing a significant effect size for both moms and dads (Table 9).

## 4. Effects from Child Attachment and Self-Regulation: A Projective Scenario of Two Parent Combined Parental Disorders

The following section will provide data and visuals from a projective scenario involving two disordered parents. Controlling for the effects of additional variables that may have moderated or mediated the influence of the IV on the DV was outside the scope of the current study because this study represents a simplistic comprehensive attempt to compare the differences in effect sizes for the occurrence of insecure attachment formation and malfunctions in self-regulation development in the context of one versus two disordered parents. As a result of aggregating all studies' effect sizes, Table 10 presents a very interesting depiction of a projective scenario that presents effects from insecure child attachment and self-regulation malfunction when reared by two disordered parents. In comparison to those effect size statistics for insecure attachment and self-regulation malfunction involving only one ill parent, the differences in effect sizes are apparent (see Tables 2, 4, and 7). At first glance, it is easy to see those studies [15, 20, 21] that yielded effects in the negative CI range, rendering the effects nonsignificant (*d* = −2.51, SE = 0.27, CI = −3.04–1.97, and *P* = 0.01; *d* = 0.23, SE = 0.14, CI = −0.04–0.49, and *P* = 0.10; *d* = 2.41, SE = 0.22, CI = −2.86–1.98, and *P* = 0.01, resp.). Many of the effect statistics were notably large and statistically significant; the three studies that were nonsignificant had little influence on the overall global effect when the studies were aggregated. Despite the three studies that produced nonsignificant effect statistics independently, the global effect was most impressive (ME/*g* + = 0.73, SE = 0.04, and *P* = 0.01).

**Figure 6:**
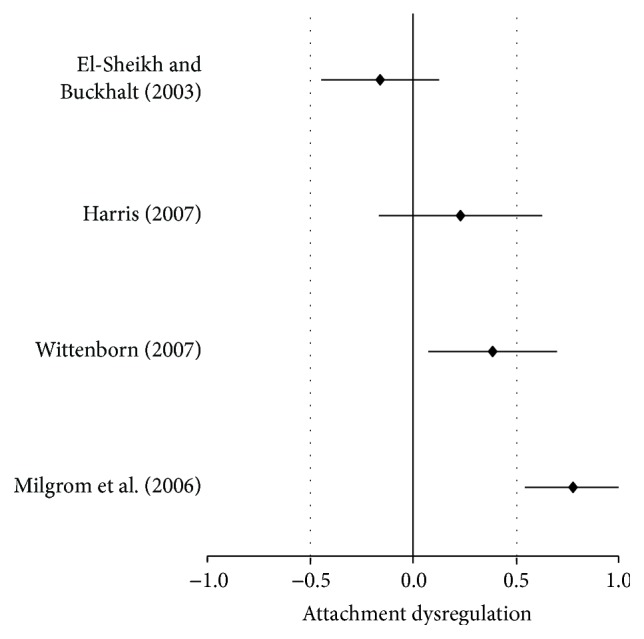
Forest plot of combined disordered parents and insecure child attachment.

**Figure 7:**
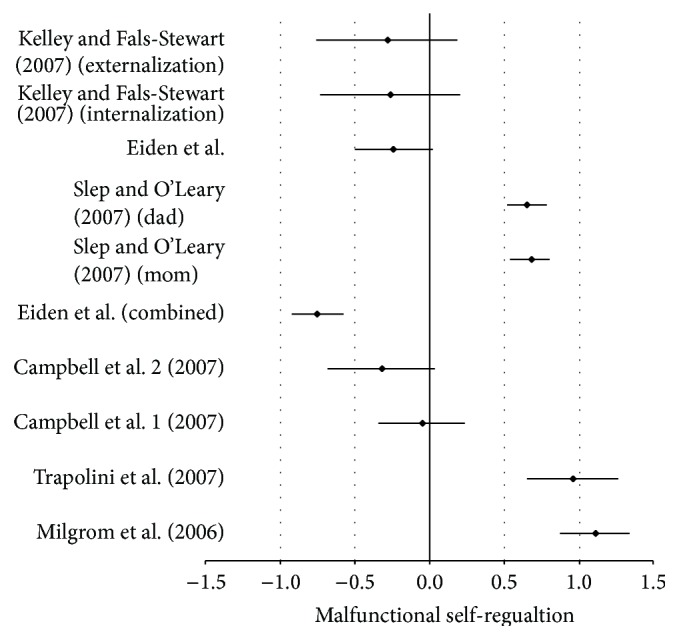
Forest plot of combined disordered parents and child self-regulation.

The notably upper range-moderate global effect (ME/*g* + = 0.73, var = 0.01, SE = 0.04, and *P* = 0.01) resulted from synthesizing the studies particularly when the reader refers back to the studies with one ill parent (Tables 3 and 6). The reader can quickly see (see Figures 6 and 7) the differences in Cohen's *d* (global effect statistics) relating to child self-regulation malfunction in comparison to one parent psychopathology from the projective scenario of two ill parents relative to the child construct (ME/*g* + = 0.56, var = 0.01, SE = 0.07, and *P* = 0.01; ME/*g* + = 0.29, var = 0.01, SE = 0.07, and *P* = 0.01, resp.).

As seen in Table 12, the *Q* statistic showed that effect sizes were heterogeneous because the test for homogeneity was clearly significant, *Q* (11) = 1109.34, critical *χ* ^2^ (11, *N* = 10), and *P* = 0.05; for example, no single effect size is representative of the collection of studies. As discussed earlier there was variability among effect sizes and this was certainly expected.

Normally with indication of significant heterogeneity, (as in Tables 1 [Table tab2] [Table tab3] [Table tab4] [Table tab5] [Table tab6] [Table tab7] [Table tab8] [Table tab9] [Table tab10] [Table tab11] [Table tab12] [Table tab13] [Table tab14] [Table tab15] [Table tab16]–17) a choice may be made to examine the distribution of effect sizes for such things as outliers or moderator or mediators that could possibly explain the variability. However, because the current study utilized a fixed effects model that assumes that effect size estimates that were combined (global effects) were estimates of the true underlying effect size [62], so the examination of the effect size distribution was not necessary or executed. The observed effect sizes from the different studies were presumed to be an estimate of the corresponding population effect of which random error occurs as a result of the “chance factors associated with subject-level sampling error in that study” ([43], page 117). This researcher simply executed a calculation of the aggregated effect sizes no matter what the source of heterogeneity in the original studies. Additionally, the researcher's only interest in conducting this study was to reveal the magnitude of the relationship, link, or association between effect size differences and the frequency of occurrence for the DVs as influenced by the IVs. Simply put, there were differences relative to larger effects sizes for insecure child attachment formation and malfunctions in self-regulation development when being reared by two disordered parents as opposed to being reared by only one disordered parent.

There were four studies total that explored child attachment formation and most of those focused on maternal depression postpartum without much paternal responsibility. The remaining six studies explored child self-regulation malfunctions and were much more inclusive of paternal influences.

## 5. Discussion

Unfortunately, studies that included paternal influences on child attachment formation were sparse at best, and those that explored child self-regulatory malfunctions do so after there is already an attachment disruption present and hence the presence of self-regulatory malfunctions. This seems to relieve any paternal obligation in terms of child attachment formation and self-regulation development, which is a major focus of the current meta-analysis as being the combined parental influence on these child constructs and not merely one parent's influence on the same. There were three studies that addressed one depressed parent and the impact on child attachment formation and self-regulation development. Upon examination and after aggregating the three studies clearly the global effect size for insecure attachment was significant for being reared by one depressed parent (*d* = 0.57, var = 0.01, SE = 0.08, and *P* = 0.01). Similarly, when examining the global effects for malfunctions in child self-regulation (*d* = 0.56, var = 0.01, SE = 0.07, and *P* = 0.01) concerning one depressed parent the results were significant as well. Unfortunately, there was only one study that addressed one alcohol disordered parent and the impact on child attachment formation; however, as an independent study the effect size was not significant (*d* = −0.16, SE = 0.15, CI = −0.45–0.13, and *P* = 0.22). Because there was only one study, the global effect (*Q* statistic) remained the same as for the one study. Upon examination of the effect sizes from one alcohol disordered parent and malfunctions in child self-regulation, the result from aggregation of the three studies was small but nonetheless significant (*d* = 0.29, var = 0.01, SE = 0.07, and *P* = 0.01). The results of the current study clearly answer the research question in which yes, there are differences in effect sizes for child attachment and self-regulation development when children are reared by two disordered parents as opposed to being reared by one disordered parent. Cohen's global effects regarding the negative implications for the child constructs from combined disordered parents were impressively larger than those global effects from one disordered parent (two parents: *d* = 0.73; one parent: *d* = 0.57, *d* = 0.56, and *d* = 0.29, resp.). It is clear that six of the 10 studies included in the current meta-analysis yielded significant effect statistics when combined thus, yielding higher effects sizes for insecurely attached children as well as higher effects for malfunctions in child self-regulation development when reared by two disordered parents than did those effects sizes on the same child constructs but reared by only one disordered parent. The current study's intent was to demonstrate that differences do exist concerning the implications to child attachment formation and subsequent self-regulation development when reared by two disordered parents as opposed to being reared by one disordered parent by way of differences in effect size statistics. As the null hypothesis was rejected by this study's data results, it is more clear that the child population being raised by two parents each with disorder(s) warrants focused exploratory research, professional acknowledgment, and broader social attention just as much as any other population under study.

The findings of the current meta-analysis indicated that there is an increased risk for the occurrence of insecure attachment formation and subsequent malfunctions in self-regulation for those children reared by two disordered patents as opposed to being reared by only one disordered parent. Having said that, future researchers may be mindful to equalize the contributions made from both parents, recognizing that each parent deserves equal attention when exploring child attachment formation and self-regulation development. Attention was noted to just how serious it is for early child development to have both parents with a psychological disorder that it posed an increased risk for negative consequences. This was evidenced by the increase in the occurrence of insecure child attachment and malfunctions in child self-regulation when reared by two disordered parents as opposed to being reared by one disordered parent.

This meta-analysis was executed because of this researcher's passion in drawing attention to the targeted population of interest, children being reared by two parents each with a psychological disorder and the implications for child attachment formation and self-regulation development. It was the intent of the current study to add validity to the notion that both parents and not just the mother or immediate caretaker are accountable for a child's attachment formation and the development of subsequent self-regulatory capabilities. For the current study, with ideas similar to Phares and Compas [63] and Connell and Goodman [64], the contention is that the paternal gap in the literature in terms of accountability for early child disruptions in attachment and malfunctions in self-regulation development has become somewhat bridged. Continuing to bridge the paternal gap is desperately needed to say the least, and by exploring the variables of interest to the present study it was obvious that a bias still exists that much of the literature reviewed was sparse in acknowledging or including paternal responsibility in the child attachment relationship.

There are substantial amounts of empirical evidence that links cognitive and behavioral deficiencies in children to parental mental illness, parental alcoholism, family dysfunction, and other biopsychosocial factors [65–67]. The problem with the existing literature is that it appeared limited in the execution of research inclusive of two disordered parents with specificity on the child constructs comprised in the current meta-analysis and relies more on the exploration of one disordered parent (normally the mother). This is especially true particularly when examining child attachment type, and because the mother is most often looked at for accountability on such a topic the paternal influence was often left out, whereas the father was mostly looked upon in terms of accountability when exploring a child's self-regulation development.

It has been this researcher's experience throughout the execution of this meta-analysis that no studies were found specifically related to combined disordered parents and the implications to child attachment formation and self-regulation development. As posed by Kelley and Fals-Stewart [21] in the literature, 20%–40% of the adults admitted to alcohol and other drug treatments had at least one child at home and that these children displayed greater problems related to self-regulation. This percentage of adults admitted to alcohol and other drug treatments that were rearing children indicates a dangerously high rate (almost 1/2 of the adult population) of adults suffering from an alcohol disorder while raising children and therefore causing increased risk to their offspring in terms of attachment formation and self-regulation development. Connell and Goodman [64] postulated that before their meta-analysis, there had not been any quantitative review of the literature pertaining to the implications of combined parental mental disturbances for early child development and thus studies that compared the effects from the same “yielded inconsistent results” (page 747). Connell and Goodman's findings suggested that the presence of a disorder in each parent presented equal risks for children even though the availability of studies for follow-up was incredibility limited. Simply put, their meta-analysis provided evidence that the risk for child developmental problems is generally as strong relative to paternal disorders as it is for maternal disorders. Therefore, it is no longer justifiable for researchers to exclude the paternal influence on any early child developmental dimension.

Connell and Goodman, among other researchers, [59, 68] draw attention to the need for researchers, professionals, and scholars to examine both disordered parents' impact on early child development and not merely focus on the mother's role. The hypotheses and research questions of the current study summarized effects size differences specifically for insecure child attachment formation and malfunctions in child self-regulatory capabilities when reared by one disordered parent versus being reared by two disordered parents. The first research question posed in this study was answered by data analysis that revealed evidence that there was a higher occurrence for insecure child attachment when children were reared by two disordered parents in comparison to being reared by only one disordered parent. The second research question was also answered by data analysis that revealed evidence that there was a higher occurrence of malfunctions in child self-regulation when children were reared by two disordered parents in comparison to being reared by only one disordered parent.

Similar to Ainsworth, Bowlby, and other supporters of attachment theory [69] (Fischer, 2007) parental practices and parental behaviors affect the type of attachment relationship children developing with their parents. Positive social change may come in the form of educating pregnant women and their partners before their babies are born that would provide awareness to potential dangers for unborn children. Then parents may choose a healthier and more informed role in their child's upbringing. Additionally, mental health treatment organizations and institutions, including substance abuse programs, may adopt curriculum and services that directly involve the family members of those already receiving services. Ideally, children and spouses could be in Rehabilitative Psychosocial Services (RPS) that embrace healthy personal awareness and responsibility.

Community outreach programs might initiate public speaking events, seminars, and lectures to educate younger adults while still in high school and college concerning the potential dangers to early child development that may in fact minimize the problem by way of awareness and may prompt prevention at individual and personal levels. By recognizing and addressing the increased risks to this underacknowledged population of children that are being raised by parents that each have a mental, mood, and or emotional condition has the potential to modify unhealthy cultural norms and would enhance parenting practices and perhaps positively alter a child's developmental trajectory.

Lastly, practicing professionals from all fields, practitioners, educators, and researchers alike may further examine the notion that children being reared by two disordered parents are at a higher risk for developmental problems than those being reared by one disordered parent, which may prompt specific prevention measures for the particular population of children in the current study. The results of the current study support positive social change aimed at broadening the attention towards this child population so that future generations may flourish. This study's data results also speak to the financial aspects of mental and physical health care costs and the shockingly high rates of parental and child mental illness [70–72] in this country, but they do not reflect the psychological, emotional, organizations, and societal costs of this study's child population.

As simplistic as this study may appear to some, its results support the contention that the population of children being reared by two disordered parents is suffering more as compared to children with only one disordered parent. Unfortunately, this particular child population seems to be underacknowledged, underresearched, and underserviced because of the limitations in research, scholarly acknowledgement, and social attention that are needed to precede focused and intentional action that need begin at the grassroots level if these kids are to receive any respite and intervention.

This study was exploratory in nature, and therefore caution should be used when drawing conclusions about the results. Some limitations do exist within this study, for instance, the fact that possible moderator influences (i.e., SES, ethnicity, culture, or geographic) were not within the scope of the current study. Future longitudinal research may shed further clarity and specificity regarding the increased risk for this child population being reared within an environment comprised of combined disordered parents. There are still many questions about the phenomena of parent(s) and child bonding, relative to child temperament, attachment, and the development of self-regulatory capacities. Questions have been left relatively unexplored and therefore unanswered within the context of this study's focus.

Another limitation of this study may have been the small number of studies included. Unfortunately as mentioned throughout this study, there were limitations concerning available research on this specific topic. More research in this area would also address other limitations in the current study such as indiscrete reporting and exclusion of demographical information and validity related to publication bias. Unavailable research relating to the implications for combined disordered parents for child attachment formation and self-regulation development may also be considered a limitation related to the generalizability to this study's findings. For example, it is possible that data results may be reduced in force by focusing exclusively on broadband syndromes of behavior problems rather than on specific childhood disorders. In fact, it is possible that the attachment model between parent and child [64] psychopathology may diversify across a spectrum of particular disorders.

In addition, the specific mental, mood, and behavioral disorders in parents and children were treated independently when realistically comorbidity of disorders is often present in both populations. This shows a need for immediate attention and a call for further research in this area is eminent. The results of the present meta-analysis have important implications for future clinical and empirical work. To the extent that the prevention measures and interventions need be suited to the specific population they intend to service, it is also necessary to at least address both parents as potential risks to this specific child population.

While the need to identify, treat, and protect these children is paramount, it may be equally beneficial for these children if their parents were treated as well and trained in healthy parenting practices. Findings from this study also suggest that mental health organizations and those mental health practitioners in the private sector may do well to implement augmented interventions that target both parents and their children, because normally the impact of adult or child psychopathology is often conceptualized and treated at the individual level. Hoefnagels et al. [73] proposed a “buddy system” (page 99) for children and adolescents that had mentally disordered parent(s) that may assist treatment professionals' with an intervention option that may increase children's overall well-being and the mental health of the entire family unit.

## Figures and Tables

**Table 1:** Estimates of effect sizes for insecure attachment from studies with one depressive disordered parent.

Study	Cohen's *d*	SE *d*	Lower CI	Upper CI	*z* value	*P* value
Milgrom et al. (2006) [13]	0.77	0.12	0.54	1.00	6.69	<0.01^*^
Wittenborn [14]	0.38	0.16	0.07	0.70	2.39	<0.02^*^
Harris [15]	0.23	0.20	−0.17	0.63	1.11	>0.22

^∗^ *P* < 0.05; SE *d* = standard error of Cohen's *d*; 95% CI = confidence interval of Cohen's *d*; *z* value = critical value.

**Table 2 tab2:** Overall (global) meta-analysis statistical test using Cohen's method.

Mean effect size *g*+	0.57
Variance	0.01
SD	0.08
*P* value	<0.01

**Table 3 tab3:** Estimates of effect size for malfunctions in self-regulation from studies with one depressive disordered parent.

Study	Cohen's *d*	SE *d*	Lower CI	Upper CI	*P* value
Milgrom et al. [13]	1.11	0.12	0.87	1.34	<0.01^*^
Trapolini et al. [16]	0.96	0.16	0.65	1.27	<0.01^*^
Campbell et al. 1 [17]	−0.05	0.15	−0.34	0.24	>0.37
Campbell et al. 2 [17]	−0.32	0.18	−0.68	−0.4	>0.09

^∗^ *P* < 0.05; SE *d* = standard error of Cohen's *d*; 95% CI = confidence interval of Cohen's *d*; *z* value = critical value.

**Table 4 tab4:** Overall (global) meta-analysis statistical test using Cohen's method.

Mean effect size *g*+	0.56
Variance	0.01
SE	0.07
*P* value	<0.01

**Table 5 tab5:** Estimates of effect size for insecure attachment from the one study with one alcohol disordered parent.

Study	Cohen's *d*	SE *d*	Lower CI	Upper CI	*P* value

El-Sheikh and Buckhalt [18, 19]	−0.16	0.15	−0.45	0.13	<0.02

^∗^ *P* > 0.05 = nonsignificance; SE *d* = standard error of Cohen's *d*; 95% CI = confidence interval of Cohen's *d*.

**Table 6 tab6:** Estimates of effect size for self-regulation malfunction for studies with one alcohol disordered parent.

Study	Cohen's *d*	SE *d*	Lower CI	Upper CI	*P* value
Eiden et al. [20]	−0.24	0.14	−0.50	0.03	>0.09
Kelley and Fals-Stewart [21] internalization	−0.26	0.24	−0.73	0.21	>0.22
Kelley and Fals-Stewart [21] externalization	−0.28	0.24	−0.76	0.19	>0.20
El-Sheikh and Buckhalt [18, 19] internalization	0.82	0.15	0.53	1.11	<0.01^*^
El-Sheikh and Buckhalt [18, 19] externalization	0.81	0.15	0.52	1.11	<0.01^*^

^∗^ *P* < 0.05; SE *d* = standard error of Cohen's *d*; 95% CI = confidence interval.

**Table 7 tab7:** Overall (global) meta-analysis statistical test using Cohen's method.

Mean effect size *g*+	0.29
Variance	0.01
SE	0.07
*P* value	<0.01

**Table 8 tab8:** Estimates of effect size for self-regulation malfunction for the two studies with combined parental psychopathology.

Study	Cohen's *d*	SE* d *	Lower CI	Upper CI	*P* value
Eiden et al. [22], mom at child age 3	0.63	0.09	0.45	0.80	<0.01^*^
Eiden et al. [22], dad at child age 3	0.63	0.09	0.45	0.80	<0.01^*^
Eiden et al. [22], mom at child age 4	−0.68	0.09	0.85	0.50	<0.01^*^
Eiden et al. [22], dad at child age 4	0.52	0.09	0.85	0.50	<0.01^*^
Eiden et al. [22], combined parent reported at child age 4	−0.75	0.09	−0.92	−0.57	<0.01^*^
Slep and O'Leary [23], mom report	0.68	0.07	0.54	0.81	<0.01^*^
Slep and O'Leary [23], dad report	0.65	0.07	0.52	0.79	<0.01^*^

^∗^ *P* < 0.05; SE *d* = standard error of Cohen's *d*; 95% CI = confidence interval.

**Table 9 tab9:** Overall (global) meta-analysis statistical test using Cohen's method.

Mean effect size *g*+	0.32
Variance	0.00
SE	0.03
*P* value	<0.01

**Table 10 tab10:** Estimates of effect size for self-regulation malfunction for combined disordered parents.

Study	Cohen's *d*	SE *d*	Lower CI	Upper CI	*P* value
Milgrom et al. [13]	2.63	0.15	2.33	2.93	<0.01
Trapolini et al. [16]	1.51	0.31	0.90	2.11	<0.01
Campbell et al. [17]	5.12	0.24	4.66	5.88	<0.01
Wittenborn [14]	2.91	0.25	2.42	3.40	<0.01
Harris [15]	2.51	0.27	3.04	1.97	<0.01
Eiden et al. [22], depressed dad	0.71	0.20	0.31	1.11	<0.01
Eiden et al. [22], depressed mom	0.52	0.20	0.12	0.91	>0.02
Slep and O'Leary [23], depressed mom	0.56	0.10	0.37	0.75	<0.01
Slep and O'Leary [23], depressed dad	0.45	0.10	0.26	0.64	<0.01
Eiden et al. [20]	0.23	0.14	0.04	0.49	>0.10
Kelley and Fals-Stewart [21]	2.42	0.22	2.86	−1.98	<0.01
El-Sheikh and Buckhalt [18, 19]	1.71	0.17	1.38	2.03	<0.01
Eiden et al. [22], dad binge drinking	3.13	0.28	2.58	3.67	<0.01
Eiden et al. [22], mom binge drinking	2.50	0.25	2.00	2.99	<0.01
Slep and O'Leary [23], mom alcoholic	0.26	0.09	0.08	0.45	<0.01
Slep and O'Leary [23], dad alcoholic	0.24	0.09	0.06	0.43	<0.01

*d* = Cohen's effect; SE *d* = standard error; 95% CI: the 95% confidence interval *d*; *P* value < 0.05.

**Table 11 tab11:** Overall (global) meta-analysis statistical test with combined disordered parents using Cohen's method.

Mean effect size *g*+	0.73
Variance	0.01
SE	0.04
*P* value	<0.01

**Table 12 tab12:** Results from the test of homogeneity for combined disordered parents for child constructs.

*Q* value	1109.34
df	15
*P* value	<0.01

**Table 13 tab13:** Estimates of effect sizes from studies with one depressive disordered parent.

Study	Cohen's *d*	SE *d*	Lower CI	Upper CI	*P* value
Milgrom et al. [13]	2.63	0.15	2.33	293	<0.01^*^
Trapolini et al. [16]	1.51	0.31	0.90	2.11	<0.01^*^
Campbell et al. [17]	5.12	0.24	4.66	5.58	<0.01^*^
Wittenborn [14]	2.91	0.25	2.42	3.40	<0.01^*^
Harris [15]	−2.51	0.27	−3.04	−1.97	<0.01^*^

*d* = effect size; SE *d*: the standard error of Cohen's *d*; 95% CI: the 95% confidence interval of Cohen's *d*; ^*^ *P* value: testing if the estimated effect (*d*) is significant for each individual study. *P* = 0.05.

**Table 14 tab14:** Global effect from studies with one depressive disordered parent.

Mean effect size *g*+	2.31
Variance	0.01
SE	0.10
*P* value	<0.01

**Table 15 tab15:** Estimates of effect sizes from studies with one alcohol disordered parent.

Study	Cohen's *d*	SE *d*	Lower CI	Upper CI	*P* value
Eiden et al. [20]	0.23	0.14	−0.04	0.49	>0.10
Kelley and Fals-Stewart [21]	−2.42	0.22	−2.86	−1.98	<0.01^*^
El-Sheikh and Buckhalt [18, 19]	1.17	0.17	1.38	2.03	<0.01^*^

*d* = effect size; SE *d*: the standard error of Cohen's *d*; 95% CI: the 95% confidence interval of Cohen's *d*; ^*^ *P* value: testing if the estimated effect (*d*) is significant for each individual study. *P* = 0.05.

**Table 16 tab16:** Global effect from studies with one alcohol disordered parent.

Mean effect size *g*+	0.23
Variance	0.01
SE	0.10
*P* value	<0.02

**Table 17:** Estimates of effect sizes from the two studies with combined disordered parents.

Study	Cohen's *d*	SE *d*	Lower CI	Upper CI	*P* value
Eiden et al. [22], dad depressed	0.71	0.20	0.31	1.11	<0.01
Eiden et al. [22], mom depressed	0.52	0.20	0.12	0.91	<0.02
Eiden et al. [22], dad binging	3.13	0.28	2.58	3.67	<0.01
Eiden et al. [22], mom binging	2.50	0.25	2.00	2.99	<0.01
Slep and O'Leary [23], mom depressed	0.56	0.10	0.37	0.75	<0.01
Slep and O'Leary [23], dad depressed	0.45	0.10	0.26	0.64	<0.01
Slep and O'Leary [23], mom alcoholism	0.26	0.09	0.08	0.45	<0.01
Slep and O'Leary [23], dad alcoholism	0.24	0.09	0.06	0.43	<0.01

*d* = effect size; SE *d*: the standard error of Cohen's *d*; 95% CI: the 95% confidence interval of Cohen's *d*; *P* value: testing if the estimated effect (*d*) is significant for each individual study. *P* = 0.05.

**Table 18:** Global effect for the two studies with combined disordered parents.

Mean effect size *g*+	0.53
Variance	0.00
SE	0.04
*P* value	<0.01

## Appendices

### A. Imputed Studies

The studies of which data were used for this meta-analysis are shown in [13–17, 19–23].

### B. Tables of Parent Data

See Tables 13, 14, 15, 16, 17, and 18.
